# Pharmaceuticals in Tap Water: Human Health Risk Assessment and Proposed Monitoring Framework in China

**DOI:** 10.1289/ehp.1206244

**Published:** 2013-05-10

**Authors:** Ho Wing Leung, Ling Jin, Si Wei, Mirabelle Mei Po Tsui, Bingsheng Zhou, Liping Jiao, Pak Chuen Cheung, Yiu Kan Chun, Margaret Burkhardt Murphy, Paul Kwan Sing Lam

**Affiliations:** 1 State Key Laboratory in Marine Pollution, and; 2 Department of Biology and Chemistry, City University of Hong Kong, Hong Kong Special Administrative Region, China; 3 The University of Queensland, National Research Centre for Environmental Toxicology, Brisbane, Australia; 4 State Key Laboratory of Pollution Control and Resource Reuse, School of the Environment, Nanjing University, Nanjing, China; 5 State Key Laboratory of Freshwater Ecology and Biotechnology, Institute of Hydrobiology, Chinese Academy of Sciences, Wuhan, China; 6 Third Institute of Oceanography, and; 7 Key Laboratory of Global Change and Marine-Atmospheric Chemistry, State Oceanic Administration, Xiamen, China

**Keywords:** China, indicator, life stage, pharmaceuticals, risk assessment, tap water

## Abstract

Background: Pharmaceuticals are known to contaminate tap water worldwide, but the relevant human health risks have not been assessed in China.

Objectives: We monitored 32 pharmaceuticals in Chinese tap water and evaluated the life-long human health risks of exposure in order to provide information for future prioritization and risk management.

Methods: We analyzed samples (*n* = 113) from 13 cities and compared detected concentrations with existing or newly-derived safety levels for assessing risk quotients (RQs) at different life stages, excluding the prenatal stage.

Results: We detected 17 pharmaceuticals in 89% of samples, with most detectable concentrations (92%) at < 50 ng/L. Caffeine (median–maximum, nanograms per liter: 24.4–564), metronidazole (1.8–19.3), salicylic acid (16.6–41.2), clofibric acid (1.2–3.3), carbamazepine (1.3–6.7), and dimetridazole (6.9–14.7) were found in ≥ 20% of samples. Cities within the Yangtze River region and Guangzhou were regarded as contamination hot spots because of elevated levels and frequent positive detections. Of the 17 pharmaceuticals detected, 13 showed very low risk levels, but 4 (i.e., dimetridazole, thiamphenicol, sulfamethazine, and clarithromycin) were found to have at least one life-stage RQ ≥ 0.01, especially for the infant and child life stages, and should be considered of high priority for management. We propose an indicator-based monitoring framework for providing information for source identification, water treatment effectiveness, and water safety management in China.

Conclusion: Chinese tap water is an additional route of human exposure to pharmaceuticals, particularly for dimetridazole, although the risk to human health is low based on current toxicity data. Pharmaceutical detection and application of the proposed monitoring framework can be used for water source protection and risk management in China and elsewhere.

Pharmaceuticals are a group of intrinsically bioactive chemicals used in humans and animals for disease treatment and prevention and growth promotion, among other purposes. These chemicals have also been regarded as environmental contaminants in recent decades because of their potential toxicity to nontarget organisms and their ubiquitous occurrence in the environment due to extensive and continuous release from sources including municipal, hospital, agricultural, and industrial effluents (Segura et al. 2009).

Potable water sources are contaminated by human and veterinary pharmaceuticals (Huerta-Fontela et al. 2008, 2011; Watkinson et al. 2009). Incomplete removal by conventional technologies (e.g., flocculation, sedimentation, and chlorination) in drinking-water treatment plants (DWTPs) has been observed, and consequently, pharmaceuticals have been detected in tap water in several developed countries at levels of usually < 100 ng/L (Segura et al. 2009). Although pharmaceutical levels in drinking water are currently unregulated, efforts have been made to include them in environmental monitoring programs. The U.S. Environmental Protection Agency (EPA) recently added 10 active ingredients to the Third Contaminant Candidate List (CCL 3) (U.S. EPA 2009a) and proposed the inclusion of seven hormones in the third Unregulated Contaminant Monitoring Regulation (U.S. EPA 2011) as reference for future amendment of drinking-water regulations. The New York Environmental Protection Department (NYCDEP) also conducted a 1-year pilot scheme for proactive monitoring of pharmaceuticals in source water (NYCDEP 2010). Provisional safety levels for pharmaceuticals in drinking water, known as drinking-water equivalent levels (DWELs), have also been derived by a few research groups based on available chronic mammalian toxicity data, minimum inhibitory concentrations, or the lowest therapeutic doses (Bruce et al. 2010; Schwab et al. 2005). Individual pharmaceuticals are expected to pose negligible human health risks in tap water based on known levels in studied regions [World Health Organization (WHO) 2012].

Data on pharmaceutical concentrations in drinking water are available for some developed countries, but relevant information in developing countries is scarce (Segura et al. 2009). In China, human and veterinary pharmaceuticals have frequently been detected in wastewater and surface waters at concentrations of generally < 1 μg/L; levels of certain compounds, such as erythromycin-H_2_O, salicylic acid, and cefalexin, have been reported to be at the high end of the values reported globally (Jiang et al. 2011; Leung et al. 2012; Wei et al. 2011), and illegal use of prohibited veterinary drugs has been detected in slaughterhouse wastewater (Shao et al. 2009). However, the human health risks of pharmaceuticals in drinking water have not been evaluated to date. This information is needed for evaluating risk management and regulation with regard to pharmaceutical contamination in China.

In recognition of these concerns, the objectives of this study were *a*) to investigate the occurrence of 32 pharmaceutically active ingredients in drinking water in 13 cities in China; *b*) to assess and prioritize the potential risks of pharmaceutical exposure in the Chinese population via drinking water based on available or newly derived DWELs, with emphasis on exposure at different life stages; and *c*) to identify and suggest possible molecular indicators for comprehensive monitoring and for risk management of pharmaceuticals in China and elsewhere.

## Methods

*Selected pharmaceuticals, sampling, and analysis*. We analyzed a total of 32 pharmaceuticals of 16 different therapeutic classes [see Supplemental Material, p. 2, Table S1 (http://dx.doi.org/10.1289/ehp.1206244)]. Because production amounts and usage patterns of pharmaceuticals in China are currently unclear and because there is no guidance provided by Chinese regulatory agencies for analyte selection, the target compounds in the present study were selected based on *a*) reported detections in wastewater and surface water in China, Vietnam, and other developed countries; *b*) representative coverage of different pharmaceutical classes (e.g., human and veterinary pharmaceuticals); *c*) potential toxicity (e.g., evidence of carcinogenicity); and *d*) existing Chinese regulations (e.g., prohibited pharmaceuticals such as nitroimidazoles).

We collected 113 household tap-water samples from 13 major Chinese cities (Beijing, Yancheng, Nanjing, Hangzhou, Shanghai, Wuhan, Changsha, Xiamen, Guangzhou, Zhuhai, Macau, Shenzhen, and Hong Kong) in the dry season (October 2010–February 2011, *n* = 67); 9 cities (excluding Beijing, Yancheng, Xiamen, Wuhan) were sampled again in the wet season (June 2011–July 2011, *n* = 46) [see Supplemental Material, p. 2, Table S2 and Figure S1 (http://dx.doi.org/10.1289/ehp.1206244)].

We collected 5 or 6 1-L replicates from private residences in each city in different urban and residential districts, stored them in polypropylene bottles covered with aluminum foil, fortified them with ascorbic acid for quenching chlorine residues, and extracted them within 48 hr of collection. The targeted pharmaceuticals were extracted with solid phase extraction methodology previously applied for sewage (Leung et al. 2012) with modifications for broadening the number of analytes, and the pharmaceuticals were quantified using high-performance liquid chromatography–tandem mass spectrometry (HPLC-MS/MS) [see Supplemental Material, pp. 3–4 (http://dx.doi.org/10.1289/ehp.1206244)].

*Derivation of DWELs and risk assessment*. For detected compounds, the acceptable daily intake (ADI) or risk-specific dose (RSD) for noncarcinogenic and carcinogenic effects, respectively, were either adopted from provisional values established in the literature or derived using toxicological, microbiological, or therapeutic approaches applied previously [Bruce et al. 2010; Schriks et al. 2010; Schwab et al. 2005; see also Supplemental Material, pp. 4–6 (http://dx.doi.org/10.1289/ehp.1206244)].

The most restrictive ADI or RSD for each pharmaceutical was converted to a DWEL based on daily ingestion rate of drinking water and body weight. To reduce the uncertainties in exposure variation among life stages, we used age-specific 95th percentile values of daily water ingestion per body weight for 12 age intervals ranging from birth to 70 years according to the U.S. EPA *Exposure Factor Handbook* [U.S. EPA 2009b; age-specific values are summarized in Supplemental Material, Table S3 (http://dx.doi.org/10.1289/ehp.1206244)] for estimating age-dependent DWELs using Equation 1:

DWEL (ng/L) = [(ADI or RSD) × RSC_DW_ × BW × 1,000] ÷ IngR_DW_, [1]

where RSC_DW_ represents the relative source contribution of acceptable dose from drinking water, assumed to be 100% for screening purposes because unintended ingestion of pharmaceuticals occurs mainly via drinking water (Cunningham et al. 2009) except for caffeine, for which 10% was used as a default factor for general contaminants (WHO 2008); 1,000 represents the unit conversion from micrograms to nanograms; and IngR_DW_ represents the daily ingestion rate of water.

We calculated an age-dependent risk quotient (RQ) for each pharmaceutical by dividing the highest concentration in tap water by its age-dependent DWEL. The individual RQs of the 12 age intervals were integrated into a lifetime RQ profile. Pharmaceuticals with RQ ≥ 1 could potentially affect human health.

## Results and Discussion

**Figure 1:**
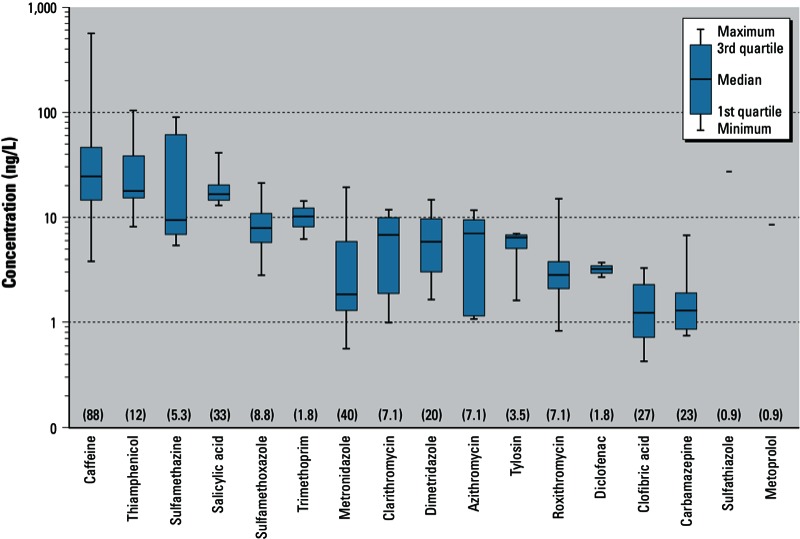
Concentrations of 17 detected pharmaceuticals in Chinese tap water. The percentage of samples with positive detections are shown in parentheses for each compound; sulfathiazole and metoprolol were only detected in a single sample each and their respective concentrations were regarded as maximum levels and shown as horizontal bars.

*Occurrence and spatiotemporal distribution*. Seventeen pharmaceuticals—including 11 human and veterinary antibiotics (macrolides, sulfonamides, thiamphenicol, nitroimidazoles, trimethoprim), 2 nonsteroidal antiinflammatory drugs (salicylic acid, diclofenac), a β-blocker (metoprolol), a lipid regulator (clofibric acid), a psychoactive stimulant (caffeine), and an anticonvulsant (carbamazepine)—were quantified (Figure 1). One or more compounds were detected in 89% of samples (calculated by dividing the number of samples containing ≥ 1 compound by the total sample number), with 92% of detectable concentrations < 50 ng/L (calculated by dividing the number of positive detections of all analytes with concentrations of < 50 ng/L by the total number of positive detections), except for caffeine, sulfamethazine, and thiamphenicol.

**Figure 2:**
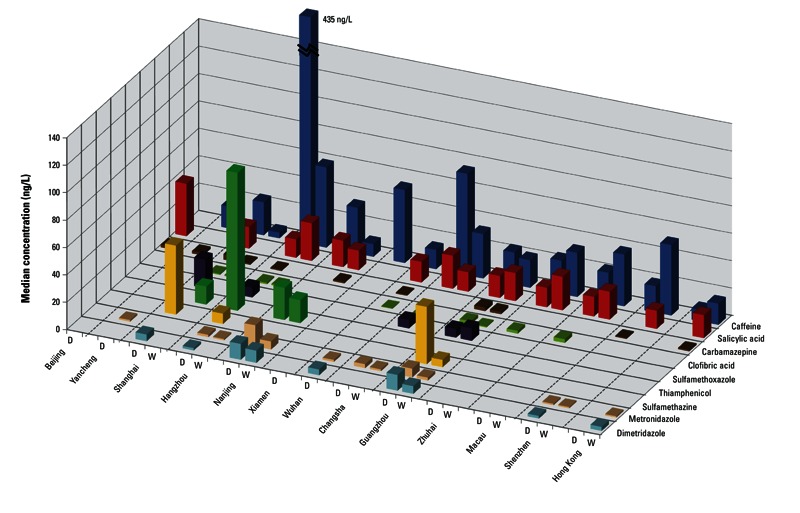
Spatiotemporal distribution of nine pharmaceuticals in Chinese tap water. Abbreviations: D, dry season; W, wet season.

Spatiotemporal distributions of nine compounds that were either detected in ≥ 10% of samples or whose maximum levels were > 50 ng/L are shown in Figure 2. Tap water collected from cities in the Yangtze River region (Nanjing and Hangzhou), together with Guangzhou and Wuhan, was found to have a detection index ≥ 3 {calculated by dividing the total number of positive detections by the number of samples in each city [see Supplemental Material, Table S4 (http://dx.doi.org/10.1289/ehp.1206244)] and concentrations}, indicating that source waters were more affected by municipal and/or agricultural wastewater. These cities should be regarded as contamination hot spots of special concern. The maximal levels of all detected compounds were found during the dry season, and six pharmaceuticals—roxithromycin, tylosin, sulfathiazole, trimethoprim, metoprolol, and diclofenac—were exclusively detected during this period. Slower river flow and lower biodegradation and photodegradation rates are the likely reasons for elevated levels in the dry season as reported for source river water in China (Jiang et al. 2011), the United States (Loraine and Pettigrove 2006), and Finland (Vieno et al. 2007).

**Figure 3:**
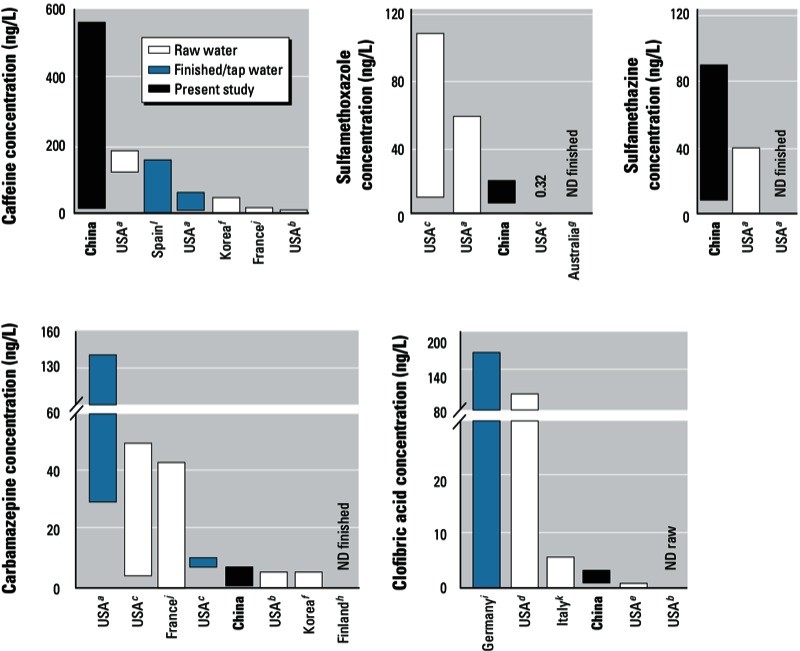
Worldwide comparison of detected pharmaceuticals in Chinese tap water. The lower and upper bounds of each box indicate median and maximum values, respectively; ND raw/finished, pharmaceutical not detected in raw/finished water. Data from ^*a*^Stackelberg et al. (2007); ^*b*^NYCDEP (2010); ^*c*^Benotti et al. (2009); ^*d*^Boyd et al. (2003); ^*e*^Loraine and Pettigrove (2006); ^*f*^Kim et al. (2007); ^*g*^Watkinson et al. (2009); ^*h*^Vieno et al. (2007); ^*i*^Heberer (2002); ^*j*^Togola and Budzinski (2008); ^*k*^Zuccato et al. (2000); ^*l*^Huerta-Fontela et al. (2008).

Human pharmaceuticals. Caffeine, which occurs both naturally and as a common food additive, was the most prevalent compound detected, occurring in ~ 88% of samples at a median concentration of 24.4 ng/L (Figure 1), but exceeding 400 ng/L (maximum, 564 ng/L) in a few samples from Hangzhou (Figure 2). In a global comparison, caffeine levels in Chinese tap water were highest among tap-water and source-water samples collected from developed countries, including the United States, Spain, and France (Figure 3). Caffeine has been identified as a marker for anthropogenic contamination in both municipal sewage and surface waters (Daneshvar et al. 2012). Its widespread occurrence in all sampled cities except Beijing revealed differing impacts of municipal sewage on source water as well as of the ineffective treatment methods currently in use in DWTPs throughout the studied regions (Figure 2). The nondetectable levels of caffeine and most of the target analytes in Beijing could be attributed to the better quality of source water {67% is groundwater [National Bureau of Statistics of China (NBSC) 2009] and also to popular utilization [49% (Beijing Waterworks Group 2008)] of advanced technologies including ozonation and activated carbon adsorption. It should be noted that these water-treatment methods are still rare in most Chinese DWTPs [see Supplemental Material, Table S2 (http://dx.doi.org/10.1289/ehp.1206244)].

Insufficient sewage treatment is a dominant factor explaining contamination of Chinese water sources. Municipal sewage is the primary source of caffeine and other human pharmaceuticals to surface waters, but only an average of 65% of sewage is conveyed to sewage treatment plants (STPs) before discharge (NBSC 2009). Indeed, up to 40% of major streams and almost all freshwater lakes have been severely impacted and classified as Type IV or below, “unsuitable for drinking purposes,” according to a national report (NBSC 2009). The DWTP is the last safeguard for preventing human exposure to chemicals in source water, but DWTPs are not designed specifically to attenuate pharmaceuticals. Among the sampled cities, 80% of tap-water supplies are treated by coagulation–sedimentation–chlorination, of which only chlorination reacts selectively with pharmaceuticals possessing electron-rich bonds such as sulfonamides and phenolic pharmaceuticals but not with caffeine and carbamazepine (Huerta-Fontela et al. 2008; Snyder et al. 2007). Ineffective attenuation of caffeine or other chemicals with similar properties during conventional treatment could account for their ubiquity in chlorinated tap water.

Unlike caffeine, other human pharmaceuticals were detected much less frequently in tap water. Salicylic acid (median–maximum, 16.6–41.2 ng/L), clofibric acid (1.2–3.3 ng/L), and carbamazepine (1.3–6.7 ng/L) were found in 23–33% of samples, followed by three macrolide antibiotics (clarithromycin, 6.7–11.9 ng/L; roxithromycin, 2.8–15.1 ng/L; azithromycin, 7.1–11.7 ng/L) and sulfamethoxazole (8.0–21.3 ng/L) in 7.1–8.8% of samples [Figure 1; see also Supplemental Material, Table S4 (http://dx.doi.org/10.1289/ehp.1206244)]. As shown in Figure 3, levels of carbamazepine, clofibric acid, and sulfamethoxazole were either comparable to or at the low end of detected values in Europe and North America. Gemfibrozil was not detected in this study, but it was present in nearly half of the treated water samples collected in the United States (7 of 18 samples; median–maximum, 0.48–2.1 ng/L; Benotti et al. 2009). This general situation was in line with results for Chinese sewage and also in surface water in Vietnam where levels of many human pharmaceuticals were also lower than studies in developed countries (Managaki et al. 2007; Sui et al. 2010), possibly because of poorer socioeconomic status and limited access to some pharmaceuticals and thus less consumption per capita in China and other low- to middle-income countries (Chen and Schweitzer 2008; WHO 2011). However, given the rapid socioeconomic development rate of China, it is reasonable to foresee that pharmaceutical usage per capita may advance to levels comparable to those in developed countries. Frequent detections of some human pharmaceuticals in the current investigation should thus be regarded as early warning signals about the current sewerage systems, STP and DWTP treatment capabilities, and water source protection in China.

Veterinary pharmaceuticals. Six veterinary pharmaceuticals—dimetridazole, metronidazole, thiamphenicol, sulfamethazine, sulfathiazole, and tylosin—were found at median concentrations of 1.8–17.8 ng/L and maximum levels of 7.0–104 ng/L [Figure 1; see also Supplemental Material, Table S4 (http://dx.doi.org/10.1289/ehp.1206244)].

The two most prevalent veterinary pharmaceuticals—dimetridazole (median–maximum, 6.9–14.7 ng/L; detection frequency, 20%) and metronidazole (1.8–19.3 ng/L; 40%)—were localized to Guangzhou, Wuhan, Changsha, and the Yangtze River region, particularly in Nanjing in Jiangsu Province, where veterinary pharmaceuticals have been reported to be widespread in surrounding surface waters (Wei et al. 2011). Metronidazole and dimetridazole have not been measured in tap water elsewhere in the world as far as we are aware. The lack of global information may be attributed to their restricted use in many developed countries because of potential carcinogenicity in mammals [Australian Pesticides and Veterinary Medicines Authority (APVMA) 2007; European Agency for the Evaluation of Medicinal Products 1997]. The United States, Canada, and Australia confine the use of dimetridazole to non–food-producing animals, and the European Union (EU) has banned veterinary administration of both compounds (APVMA 2007; EU 2010). In contrast, use of these two nitroimidazoles is authorized for therapy in food-producing animals in China but detectable residue levels in food commodities are prohibited (Ministry of Agriculture of The People’s Republic of China 2002). Our findings show that tap water would be an additional route of human exposure in China to these prohibited veterinary drugs other than via food ingestion, potentially increasing human health risk.

Sulfamethazine and thiamphenicol were only found in 5.3% and 12% of samples, but their respective maximum levels reached 89.6 ng/L in Shanghai and 104.3 ng/L in Hangzhou [see Supplemental Material, Table S4 (http://dx.doi.org/10.1289/ehp.1206244)]. Sulfamethazine was not detected in raw water in a 1-year monitoring study in the United States and the maximum concentration in raw water (40 ng/L) in another U.S. study was only half of that detected in the present study (NYCDEP 2010; Stackelberg et al. 2007) (Figure 3). Shanghai’s water source, the Huangpu River, has been deteriorated by STP and animal husbandry effluents, and it has been reported to contain sulfamethazine at a maximum level of 623 ng/L (Jiang et al. 2011). Substantially higher detection frequencies and environmental levels of sulfamethazine were found in Jiangsu Province in China [detected in 88% of 18 samples, median–maximum, 100–4660 ng/L (Wei et al. 2011)] and in Hanoi, Vietnam [100% of 20 samples, 81–328 ng/L (Managaki et al. 2007)], compared with a national reconnaissance of pharmaceuticals in streams across the United States, which reported a few detections of sulfamethazine at low levels [4.8% of 104 samples, 20–120 ng/L (Kolpin et al. 2002)]. Veterinary pharmaceutical contamination in drinking-water sources could be a characteristic problem in China and/or other developing countries because of the combination of extensive agribusiness and resultant large-scale release of veterinary medicines from animal breeding farms with inadequate waste treatments as well as agricultural surface runoff (Jiang et al. 2011; Managaki et al. 2007; Tong et al., 2009; Wei et al. 2011). Moreover, poor source-water quality could affect treatment efficiencies in DWTPs. Given the substantial reactivity of the aniline moiety in sulfonamides during chlorination, their presence in tap water was unexpected. High levels of coexisting sewage-derived chemicals such as ammonia may compete for free chlorine and thus interfere with expected treatment efficiency (Dodd and Huang 2004).

*Human health risk assessment.* Previous risk assessments of pharmaceuticals in drinking water have employed different approaches, but life-stage–specific exposure was not considered, other than applying default values of body weight and drinking-water rate for adults (70 kg; 2 L/day) and for children (10 or 14 kg; 1 L/day) (U.S. EPA 2008). We attempted to reduce uncertainty in the exposure assessment by integrating different age-specific exposure factors for evaluating life-stage risks. The current approach is more conservative and can be modified further by incorporation of age-specific adjustment factors if a pharmaceutical is known to be particularly toxic to a certain life stage according to variability in toxicokinetics and toxicodynamics (Daston et al. 2004; Ginsberg et al. 2004). However, one limitation of the present study is that there is no detailed information about drinking-water consumption rates and body weights of the Chinese population, and thus data from the U.S. EPA *Exposure Factors Handbook* (U.S. EPA 2009b) was used as the basis for risk assessment. Nevertheless, the current assessment can be considered a proactive human health risk assessment of pharmaceuticals in Chinese tap water and a reference for future risk management in China.

**Figure 4:**
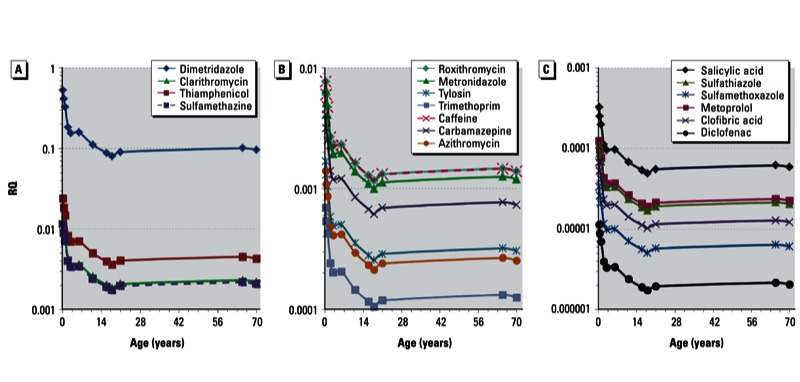
Human health life-stage RQ profile of detected pharmaceuticals in Chinese tap water. (*A*) Pharmaceuticals with at least one life-stage RQ ≥ 0.01. (*B*) Pharmaceuticals with all life-stage RQs between 0.0001 and 0.01. (*C*) Pharmaceuticals with most life-stage RQs < 0.0001.

The most restrictive ADIs or RSDs for the detected pharmaceuticals ranged from 0.006 to 150 μg/kg/day for different health end points [see Supplemental Material, Table S3 (http://dx.doi.org/10.1289/ehp.1206244)]. Pharmaceuticals were categorized into three groups: *a*) Group 1, at least one life-stage RQ ≥ 0.01; *b*) Group 2, 0.01 > all life-stage RQs ≥ 0.0001; *c*) Group 3, most life-stage RQs < 0.0001, and lifetime RQ profiles from birth to 70 years of age were plotted (Figure 4). Generally, life-stage RQs of the 17 assessed pharmaceuticals for the 12 age intervals ranged from 0.5 to < 0.0001, of which 13 were either in Group 2 or 3, and thus were at least 2 orders of magnitude lower than their DWELs (see Supplemental Material, Table S3). Among the assessed life stages, RQs in infants (birth to < 12 months) and children (age 1 to < 11 years) were at least 1.2–5.8 times greater than the approximately constant RQs throughout adolescence and adulthood (Figure 4). Greater drinking-water ingestion on a body-weight basis in these early life stages could account for higher exposure levels and thus higher risks than in adults.

Three veterinary medicines (dimetridazole, thiamphenicol, and sulfamethazine) and a human pharmaceutical (clarithromycin) were classified into Group 1. The DWELs of clarithromycin, thiamphenicol, and sulfamethazine were derived based on inhibition of intestinal microbes, hemotoxic effects, and potential incidence of thyroid gland follicular adenoma, respectively [see Supplemental Material, Table S3 (http://dx.doi.org/10.1289/ehp.1206244)]; these Group 1 compounds exceeded their respective criterion only for a relatively short period (< 1 year) after birth. As the relevance of the toxicological end points used for DWEL derivation to infant health is unclear, further investigation of the effects of early-life exposure to these three compounds is needed to determine their potential risks. It should be noted that dimetridazole, a potential carcinogen and a prohibited residue in food commodities in China, presented the highest risks in tap water, although all life-stage RQs (0.08–0.53) were < 1. The maximum environmental concentration of dimetridazole (14.7 ng/L) was on the same order of magnitude as its age-dependent DWELs (27.8–184 ng/L). The current screening assessment considered the RSC of ADI from tap-water ingestion to be 100%, but the possibility of 20–80% contribution from tap water was also estimated (Bruce et al. 2010). In the conservative case of RSC equal to 20%, life-stage RQs of dimetridazole (0.40–2.65) increase by five times and it is potentially risky (RQ > 1) to infants for their first year of life. The end point used for DWEL derivation was the incidence of benign mammary tumors in rats under chronic exposure [no slope factor was determined; no observable adverse effect level = 4,200 μg/kg/day, average value of both sexes, was thus regarded as the maximum tolerated dose applied (Lowe et al. 1976; see also Supplemental Material, pp. 4–6, Equation S3)]. Childhood exposure to potential carcinogens is of particular concern because of children’s immature defense systems and rapid growth rate (U.S. EPA 2005). In addition, children also have a longer time frame for developing chronic diseases such as cancer initiated in any critical window early in their lives (Landrigan et al. 2004). As a result, risk management of this compound should be of first priority. Nevertheless, our results affirm those reported in the literature and internationally that appreciable risks of most individual pharmaceuticals in tap water to human health are unlikely based on available toxicity data (Schwab et al. 2005; WHO 2012).

Although all of the detected pharmaceuticals posed low risk when considered individually, we also considered potential uncertainties in the analysis. One critical issue identified in a recent summit was that mixture toxicities and possible interactions of these biologically active xenobiotics are not yet well understood, in particular chronic exposure to trace amounts of pharmaceutical mixtures and their corresponding metabolites (Rodriguez-Mozaz and Weinberg 2010). Moreover, given that unique prenatal and early-life susceptibility to pharmaceuticals during critical windows of development may result in unanticipated adverse effects, there is also a need to evaluate exposures to pharmaceuticals in tap water *in utero* and through breastfeeding in future life-stage assessments (Landrigan et al. 2004).

*Risk management*. China is currently lacking a regulatory framework for preventing and mitigating the occurrence of human and veterinary pharmaceuticals in tap water. The water safety plan approach suggested by the WHO (2009) includes 11 modules and can be used as a backbone for targeting these emerging contaminants. Of the modules suggested by the WHO, we have focused on the following elements or stages:

Hazards/targets identification and desktop-based prioritization
Screening, risk assessment, and risk-based prioritization
Comprehensive monitoring program
Planning and implementation of mitigation measures and follow-up reassessment.

**Figure 5:**
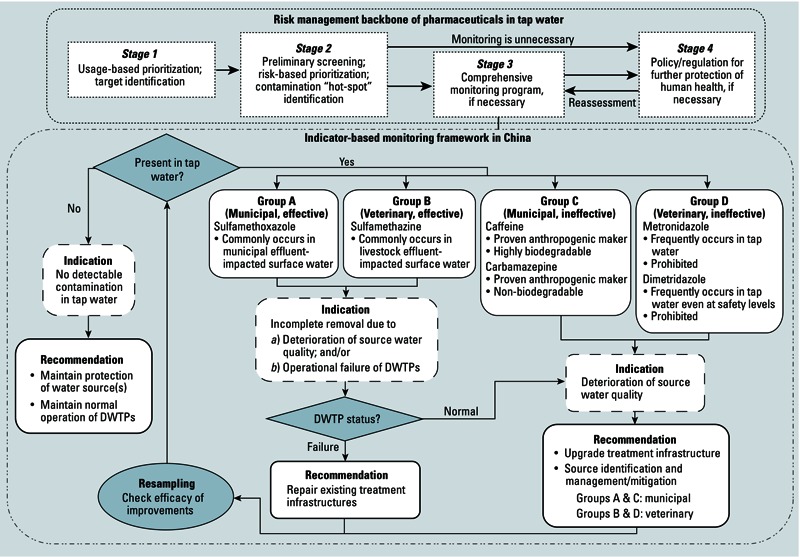
Risk management and indicator-based monitoring framework for pharmaceuticals in Chinese tap water.

The WHO approach is not specific to any group of contaminants or water quality parameters. Here, we have applied this general approach to establish a monitoring and risk assessment framework for pharmaceuticals in tap water (Figure 5). In the case of countries where usage information is lacking, a semiquantitative estimation based on surveys of hospitals and practitioners could be an alternative information source in stage 1, or large-scale screening could be implemented in stage 2 if pharmaceutical contamination in raw and tap water is reasonably anticipated based on existing evidence. In this study, we applied the latter approach and identified locations and contaminants of concern in Chinese tap water for management prioritization.

Stages 3 and 4 of our framework involve intensive time, cost, and human resources, and the need for these efforts should be considered thoughtfully according to risk levels. Given the low risks currently posed by most of the measured pharmaceuticals in Chinese tap water, a routine national monitoring program and installation of specialized treatment infrastructure are not immediately warranted. However, regions with elevated levels (e.g., cities within the Yangtze River region) or frequent detection of potentially risky compounds (e.g., dimetridazole) require the application of a proactive monitoring framework based on a suite of indicator compounds with scenario-based interpretations and recommendations (Figure 5). In the present study, an indicator is regarded as a qualitative measure to reflect contamination by micropollutants with similar sources and behaviors under different treatments and is also used to evaluate the efficacy of DWTP treatment (Dickenson et al. 2009). Indicators for micropollutants described in the literature focused exclusively on municipal wastewater-derived compounds (e.g., caffeine, carbamazepine) (Gasser et al. 2010), but the frequent detection of both municipal and animal wastewater-derived pharmaceuticals suggests that corresponding indicators for both origins are particularly necessary in China and perhaps other developing countries. Therefore, we selected six indicators according to *a*) origin specificity, in which the indicator exclusively originates from either municipal or veterinary wastewater; *b*) behavior in DWTPs, in which the treatability of pharmaceuticals is classified based on chlorination, the most popular treatment method in China, but indicators for other advanced treatments (e.g., ozonation) could be further developed (Dickenson et al. 2009); *c*) representativeness, in which the indicator should be commonly detected and at relatively higher concentrations. All of the selected indicators are categorized into four groups based on origin and removal efficiency by chlorination in DWTP (Figure 5):

Group A (municipal, effective): sulfamethoxazole
Group B (veterinary, effective): sulfamethazine
Group C (municipal, ineffective): caffeine and carbamazepine
Group D (veterinary, ineffective): dimetridazole and metronidazole).

Because the indicator in groups A and B is susceptible to chlorination, its presence in tap water may qualitatively indicate ineffective chlorination treatment and/or poor quality of raw water from which the indicator is not completely removed even under normal operating conditions; immediate verification of the operating conditions in the DWTP would therefore be suggested. Indicators in groups C and D directly reflect the water quality of raw water, and their presence may denote that water sources are negatively impacted. Upgrading current treatment technologies is a possible mitigation measure, but controlling pharmaceutical inputs at contamination sources is also highly recommended. Origins of impacts could be traced back and then further mitigated in terms of short-term measures (e.g., changes of effluent discharge points, relocations of nearby point sources, strengthening enforcement of potable water source protection) and also long-term regulations (e.g., drug take-back programs, changes in pharmaceutical disposal practices, raising public awareness by education) (WHO 2012).

This indicator-based framework provides *a*) cost- and time-effective monitoring instead of requiring quantification of a broad range of pharmaceutical compounds; *b*) clues for tracing and identifying potential contamination sources in raw water; *c*) insights into monitoring treatment effectiveness; and *d*) a reference for assisting regulatory authorities in decision making related to risk minimization and public health protection policies. This framework has been developed for Chinese tap water according to the results of the present study, but it can be more widely applied. Selection of indicators will likely vary among countries based on patterns of pharmaceutical usage, DWTP treatment levels, and analytical capability; a relatively large-scale initial survey of pharmaceutical levels and patterns is therefore still needed for indicator identification. Further development of the current indicator-based framework (e.g., broadening the number of suitable indicators to reflect other DWTP treatment types and effects of in-stream transportation) should be carried out for more a comprehensive and informative assessment of pharmaceutical risk in tap water.

## Supplemental Material

(1.1 MB) PDF
