# Supplementary material for: Pharmaceuticals in Tap Water: Human Health Risk Assessment and Proposed Monitoring Framework in China
Source: Environ Health Perspect. 2013 May 10;121(7):839–46. doi: 10.1289/ehp.1206244 (PMC3702003; doi:10.1289/ehp.1206244)
Supplement: (1.1 MB) PDF [file ehp.1206244.s001.pdf]

Supplemental Material

Pharmaceuticals in Tap Water: Human Health Risk Assessment and Proposed Monitoring Framework in China

Ho Wing Leung[1,2,*], Ling Jin[1,2,3], Si Wei[4], Mirabelle Mei Po Tsui[1,2], Bingsheng Zhou[5], Liping Jiao[6,7], Pak Chuen Cheung[1,2], Yiu Kan Chun[1,2], Margaret Burkhardt Murphy[1,2], and Paul Kwan Sing Lam[1,2]

Table of Contents

METHODS ....

Selection of pharmaceuticals ....

Sampling ....

Analysis ....

Quality assurance/quality control ....

Derivation of DWELs and risk assessment ....

Supplemental Material, Table 1. List of targeted pharmaceuticals and information about instrumental analysis and QA/QC parameters. ....

Supplemental Material, Table S2. Information for the 13 sampled cities in China..................... 1

Supplemental Material, Figure S1. Locations of the 13 sampled cities in China........................ 1

Supplemental Material, Table S3. Information about risk assessment for age-specific exposure scenarios and derivation of ADI/RSD and DWEL values.. 1

Supplemental Material, Table S4. Occurrence (ng/L) and spatiotemporal distribution of 17 detected pharmaceuticals. 1

References.. 1

METHODS

Selection of pharmaceuticals

We analyzed a total of 32 pharmaceuticals of 16 different therapeutic classes including 9 subclasses of antibiotics (viz. penicillins, cephalosporins, macrolides, sulfonamides, (fluoro)quinolones, amphenicols, nitroimidazoles, lincosamides, diaminopyrimidines), β-blockers, anti-hypertension drugs, diuretic drugs, lipid regulators, psychoactive stimulants, anticonvulsants and non-steroidal anti-inflammatory drugs (NSAIDs) (Supplemental Material, Table S1).

Sampling

The sampled cities were categorized into 4 groups according to their geographical locations: i) northern China: Beijing and Yancheng; ii) Yangtze River region: Nanjing, Hangzhou and Shanghai; iii) middle-southern China: Wuhan, Changsha and Xiamen; iv) Pearl River region in southern China: Guangzhou, Zhuhai, Macau, Shenzhen, and Hong Kong. Surface water is the dominant potable water source in the selected cities (>90% of total water supply), except for Beijing, which relies mainly on groundwater (67%) (NBSC 2009). Coagulation, sedimentation and chlorination are the most common processes in DWTPs but 18-49% of the water supplies in some sampled cities are further treated by ozonation, (bio-)activated carbon, and biofiltration (see Supplemental Material, Figure S1 and Table S2). We focused on household samples in relatively well-developed and densely-populated cities as pharmaceutical exposure could affect large populations in these locations. We also tried to maximize the geographical coverage of the samples by collecting tap water from areas with different water sources and treatment technologies under the constraint that samples had to be analyzed within 48 hours.

Analysis

The targeted pharmaceuticals were extracted with solid phase extraction methodology previously applied for sewage (Leung et al. 2012) with modifications for broadening the number of analytes and utilizing 9 isotopically-labeled standards instead of only ^{13}C-caffeine for reducing analytical uncertainties. Briefly, 500 mL of each sample was combined with 5 mL 5% (w/v) EDTA, acidified to pH 3-3.3 and then loaded on Hydrophilic-Lipophilic Balanced (HLB) cartridges preconditioned by methanol and water. After loading, the cartridge was rinsed with water and eluted with 4 mL methanol. The eluate was reduced to near-dryness (<0.1 mL) under a gentle stream of nitrogen, reconstituted to 0.5 mL with water and then centrifuged at 9000 rpm for 10 min. The final extract was spiked with 62.5 ng ^{13}C-phenacetin, 100 ng $^{13}C_3$-ibuprofen and $^{13}C_3{}^{15}$N-ciprofloxacin, and 50 ng of each remaining internal standard in order to compensate for matrix effects during instrumental quantification. For matching internal standards with analytes, we followed the quantitative methods applied in Gros et al. (2009) with slight amendments in order to minimize matrix effects. First, the slope difference of two calibration curves separately constructed in Milli-Q water and in tap water extract was calculated for each analyte. This difference was regarded as a matrix-induced interference factor and we then selected an appropriate internal standard for instrumental quantification in order to minimize the factor as close as 0 as possible. If the analyte was subject to limited matrix effects and the external calibration curve alone was the best quantification method, no internal standard was assigned. A 10 µL aliquot of extract was injected into an Agilent 1100 HPLC system (Palo Alto, CA, USA) and chromatographic separation was performed using an XBridge™ C18 column (2.1 x 50 mm, 5 µm, Waters Corporation). Analytes were ionized in electrospray ionization (ESI) source operated in positive and negative modes. Two mass transitions of each parent compound were

monitored by an ABSciex 2000 QTRAP triple quadrupole tandem mass spectrometer (MS/MS) (Toronto, Canada) for quantification and confirmation in multiple reaction monitoring (MRM) mode except ibuprofen and the mass-labeled internal standards. Quantification was carried out by normalizing analyte peak area by the corresponding internal standard peak area in sample extracts and substituting into the linear equation of a seven-point external calibration curve (0-400 μg/L) constructed in Milli-Q water.

Quality assurance/quality control

Each individual sample was accompanied by a corresponding field blank (pure water fortified with ascorbic acid) and procedural blanks (n = 15) were analyzed with each sample batch. We found no background contaminations during sample collection, transportation and analysis. The matrix-matched limit of quantification (LOQ) was defined as the sum of the average and ten times the standard deviation of all procedural blank values and then corrected by the degree of matrix effects (Leung et al. 2012). LOQs ranged from 0.2 to 26.1 ng/L. Matrix-spiked absolute recoveries (n = 25, at 100 ng/L) ranged from 64.4% to 105%, with relative standard deviations mostly lower than 20% (Supplemental Material, Table S1).

Derivation of DWELs and risk assessment

The acceptable daily intake (ADI) or risk-specific dose (RSD) were derived using toxicological, microbiological or therapeutic approaches applied previously (Bruce et al. 2010; Schriks et al. 2010; Schwab et al. 2005).

For non-cancer effects, the no-observable-adverse-effect level (NOAEL) or lowest-observable-adverse-effect level (LOAEL) for different toxicity endpoints such as developmental and reproductive effects in humans or other mammals was extrapolated to an ADI

by using equation S1, which includes five types of uncertainty factors (UFs): (UF1) extrapolation from LOAEL to NOAEL; (UF2) duration of exposure; (UF3) interspecies variation; (UF4) intraspecies variation; and (UF5) data quality (Schwab et al. 2005):

$$ADI\ (\mu g/kg \cdot d) = (NOAEL\ or\ LOAEL)/(UF1 \times UF2 \times UF3 \times UF4 \times UF5) \qquad [S1]$$

The values and considerations of each uncertainty factor were consistent with those recommended by the U.S. EPA and in recent literature (U.S. EPA 2002; Schwab et al. 2005).

Carcinogenicity risk was assessed using slope factors (SFs), referring to the tumorigenic risk per increment of dose, of a linear non-threshold dose-response curve of the observed data extrapolated to a RSD associated with an incremental lifetime cancer risk of 10^{-6} (equation S2).

$$RSD\ (\mu g/kg \cdot d) = SF/1 \times 10^{-6} \qquad [S2]$$

This approach assumes that the entire range of human variation is taken into consideration and can protect public health at low doses (U.S. EPA 2005). If only evidence of carcinogenicity but no tumor incidence data was obtained from toxicity tests, a virtually safe dose (equivalent to ADI) was estimated based on the maximum tolerated dose (MTD) determined in a 90-day bioassay study corresponding to an incremental cancer risk of 10^{-6} (Gaylor and Gold 1998; Bruce et al. 2010) (equation S3):

$$ADI\ (\mu g/kg \cdot d) = MTD/740000 \qquad [S3]$$

For antibiotics, a microbiological ADI was also derived from MICs for the most sensitive human intestinal flora using equation S4 (Bruce et al. 2010; Schwab et al. 2005):

$$ADI\ (\mu g/kg \cdot d) = (MIC_{50} \times MCC)/(FA \times SF \times BW) \qquad [S4]$$

where MIC_{50} is the concentration inhibiting 50% of strains; MCC is the mass colonic content = 220 g/d; FA is the fraction of the oral dose available to microorganisms in the intestines; SF is the safety factor, normally equal to 1 if MIC_{50} data is adequate; and BW is body weight = 60 kg (approximately average between Chinese female: 57 kg; and male: 66 kg; based on a marketing survey in China, Alvanon 2008).

If toxicological and microbiological data were deficient for a given compound, the lowest therapeutic dose was regarded as the LOAEL for derivation of a therapeutic ADI (Schwab et al. 2005).

Supplemental Material, Table S1. List of targeted pharmaceuticals and information about instrumental analysis and QA/QC parameters.

				Precursor	Transition 1			Transition 2			QA/QC parameters	
Therapeutic class	Pharmaceutical	Supplier	Retention time (min)	(*m/z*)	(*m/z*)	DP (V)	CE (V)	(*m/z*)	DP (V)	CE (V)	LOQ (ng/L)	Recovery (%) Mean ± RSD
Penicillins	Ampicillin[a]	Sigma-Aldrich	6.47	350.5	106.0	42	35	160.0	42	18	3.7	74.5 ± 9.5
Cephalosporins	Cefalexin[a]	Riedel-de Haën	6.07	348.1	158.3	66	15	174.2	66	17	3.7	72.2 ± 9.5
	Cefotaxime[b]	Fluka	6.37	454.2	239.0	-51	-16	394.1	-36	-10	3.4	85.2 ± 10.5
	Cefuroxime[b]	Dr. Ehrenstorfer[c]	6.55	423.2	207.0	-40	-20	318.0	-40	-15	2.2	64.4 ± 17.2
Macrolides	Clarithromycin[d]	Sigma-Aldrich	10.50	748.8	590.3	76	31	558.5	76	31	0.7	81.2 ± 13.3
	Roxithromycin[d]	Sigma-Aldrich	10.60	837.8	158.5	41	45	679.8	36	31	0.3	69.3 ± 12.0
	Azithromycin[d]	Fluka	10.50	749.0	158.0	65	53	591.4	65	53	0.3	80.0 ± 13.8
	Tylosin[e]	Sigma-Aldrich	9.71	916.3	174.1	96	53	772.3	81	53	0.9	72.7 ± 11.8
Sulfonamides	Sulfathiazole[f]	Sigma-Aldrich	2.98	256.0	156.0	46	17	108.0	41	17	3.7	84.6 ± 7.4
	Sulfamethazine[f]	Sigma-Aldrich	5.23	279.1	186.1	76	21	124.1	56	35	3.3	90.5 ± 18.4
	Sulfamethoxazole[a]	Sigma-Aldrich	6.38	254.1	156.0	66	17	108.0	71	35	2.7	79.4 ± 15.0
Fluoroquinolones	Norfloxacin[g]	Sigma-Aldrich	6.11	320.2	302.2	51	30	276.0	46	17	21.3	105 ± 17.6
	Flumequine[d]	Sigma-Aldrich	9.54	262.4	202.1	30	45	244.2	30	28	14.4	86.5 ± 20.5
Amphenicols	Chloramphenicol[b]	Riedel-de Haën	7.81	321.0	152.0	-71	-22	257.1	-76	-12	2.1	98.2 ± 19.6
	Thiamphenicol[h]	Sigma-Aldrich	4.80	354.0	185.0	-45	-26	79.0	-80	-45	5.2	86.9 ± 14.4
Nitroimidazoles	Dimetridazole[i]	Dr. Ehrenstorfer	2.08	142.1	96.3	43	20	81.2	43	37	1.5	92.8 ± 13.5
	Metronidazole[j]	Dr. Ehrenstorfer	1.79	172.2	128.0	40	20	82.0	40	32	0.4	91.8 ± 19.5
Lincosamides	Lincomycin[e]	Fluka	4.71	407.0	126.2	50	40	359.4	50	30	0.2	80.9 ± 10.5
	Clindamycin[d]	Sigma-Aldrich	8.77	425.0	126.3	45	40	377.3	45	40	0.3	87.9 ± 13.6

Therapeutic class	Pharmaceutical	Supplier	Retention time (min)	Precursor	Transition 1			Transition 2			QA/QC parameters	
				(*m/z*)	(*m/z*)	DP (V)	CE (V)	(*m/z*)	DP (V)	CE (V)	LOQ (ng/L)	Recovery (%) Mean ± RSD
Diaminopyrimidines	Trimethoprim[i]	Sigma-Aldrich	5.36	291.2	123.0	66	33	261.2	71	23	5.2	89.7 ± 12.5
β-blockers	Metoprolol[f]	Sigma-Aldrich	6.78	268.0	121.0	51	32	133.0	46	33	4.1	98.2 ± 8.9
	Acebutolol[f]	Sigma-Aldrich	6.86	337.5	116.0	41	29	72.0	41	43	1.5	100 ± 10.7
Anti-hypertensive drug	Enalapril[d]	Sigma-Aldrich	8.75	377.7	234.2	43	27	303.3	43	27	0.5	91.9 ± 8.0
Diuretic drug	Hydrochlorothiazide[b]	Sigma-Aldrich	2.28	296.1	78.1	-80	-41	268.9	-80	-30	7.8	88.0 ± 15.5
Lipid regulators	Clofibric acid[d]	Dr. Ehrenstorfer	11.30	213.0	127.2	-34	-23	84.9	-34	-16	0.4	96.5 ± 10.1
	Gemfibrozil[d]	Sigma-Aldrich	13.50	249.2	121.1	-45	-25	127.4	-45	-15	1.3	84.9 ± 9.5
Psychoactive stimulant	Caffeine[j]	Fluka	5.75	195.3	138.0	40	28	110.0	40	30	3.8	88.7 ± 7.4
Antiepileptic drug	Carbamazepine[d]	Sigma-Aldrich	9.73	237.3	194.1	53	30	179.2	53	50	0.7	93.8 ± 8.9
Non-steroidal anti-inflammatory drugs (NSAIDs)	Diclofenac[d]	Sigma-Aldrich	12.51	294.0	250.0	-25	-12	214.0	-25	-25	1.2	86.1 ± 7.9
Non-steroidal anti-inflammatory drugs (NSAIDs)	Naproxen[b]	Sigma-Aldrich	11.40	229.2	169.0	-30	-42	170.0	-30	-24	2.7	74.5 ± 20.8
	Ibuprofen[d]	Sigma-Aldrich	12.80	205.0	161.0	-40	-16				16.2	89.4 ± 15.0
	Salicylic acid[b]	Sigma-Aldrich	8.48	137.1	93.1	-30	-25	65.2	-30	-40	13.0	73.0 ± 20.9
Internal standards	$^{13}C_2$-Erythromycin-H_2O	CIL[k]	10.70	719.0	160.0	71	38					
	$^{13}C_6$-Sulfamethoxazole	CIL	6.37	260.3	98.2	45	40					
	$^{13}C_3{}^{15}N$-Ciprofloxacin	CIL	6.25	336.5	318.0	56	34					
	$^{13}C_3$-Trimethoprim	CIL	5.33	294.5	126.0	60	35					
	$^{13}C_3$-Caffeine	CIL	5.58	198.1	140.1	61	19					
	D_5-Chloramphenicol	CIL	7.73	326.2	157.0	-60	-24					
	$^{13}C_3$-Ibuprofen	CIL	12.80	208.0	163.0	-26	-16					

				Precursor	Transition 1			Transition 2			QA/QC parameters	
**Therapeutic class**	**Pharmaceutical**	**Supplier**	**Retention time (min)**	**(*m/z*)**	***(m/z)***	**DP (V)**	**CE (V)**	***(m/z)***	**DP (V)**	**CE (V)**	**LOQ (ng/L)**	**Recovery (%) Mean ± RSD**
	^{13}C-Phenacetin	Sigma-Aldrich	8.05	181.3	110.2	60	29					
	D_3-Mecoprop	Dr. Ehrenstorfer	11.80	216.0	144.0	-30	-20					

[a] $^{13}C_6$-Sulfamethoxazole; [b] $^{13}C_3$-Ibuprofen; [c] Dr. Ehrenstorfer GmBH, Augsburg, Germany; [d]External calibration curve applied for quantification; [e] $^{13}C_2$-Erythromycin-H_2O; [f] ^{13}C-Phenacetin; [g] $^{13}C_3{}^{15}N$-ciprofloxacin; [h] D_3-mecoprop; [i] $^{13}C_3$-trimethoprim; [j] $^{13}C_3$-caffeine; [k]Cambridge Isotope Laboratory Inc.

Supplemental Material, Table S2. Information for the 13 sampled cities in China.

	City	Province	Population ($1x10^4$)	Water supply ($1x10^4$ m^3/day)	Treatment process[d]
A	Beijing	DCM[a]	1961	343[c]	Conv, 49% O_3+(Bio)ActC
B	Yancheng	Jiangsu	159[b]	23.5[b]	Conv
C	Nanjing	Jiangsu	771	220	Conv
D	Shanghai	DCM[a]	1921	577[b]	Conv, 21% O_3+(Bio)ActC
E	Hangzhou	Zhejiang	429[a]	170	Conv, 41% O_3+(Bio)ActC
F	Wuhan	Hubei	500[a]	375[b]	Conv
G	Changsha	Hunan	362[a]	165	Conv, 18% O_3+(Bio)ActC
H	Xiamen	Fujian	252	121	Conv
I	Guangzhou	Guang-dong	887	465	Conv, 22% O_3+(Bio)ActC
J	Zhuhai	Guang-dong	145	52	Conv
K	Macau	SAR[a]	54	33	Conv
L	Shenzhen	Guang-dong	891[b]	376	Conv, 38% O_3+(Bio)ActC
M	Hong Kong	SAR[a]	710	261	Conv, 21% Biofil/ O_3+Biofil

See Supplemental Material, Figure S1 for map showing the location of each city.

[a] DCM: Directly-controlled municipality; SAR: Special Administrative Region; [b] Refers to the metropolitan area of the city

[c] Beijing: 65% from groundwater; Other cities: >90% from surface water; [d] Percentage of raw water treated by non-conventional treatment. Conv (Conventional treatment): coagulation + flocculation + sedimentation + chlorination; O_3: ozonation; (Bio)ActC: (bio)activated carbon adsorption; Biofil: biofiltration; percentage of water supply treated by treatments other than chlorination.

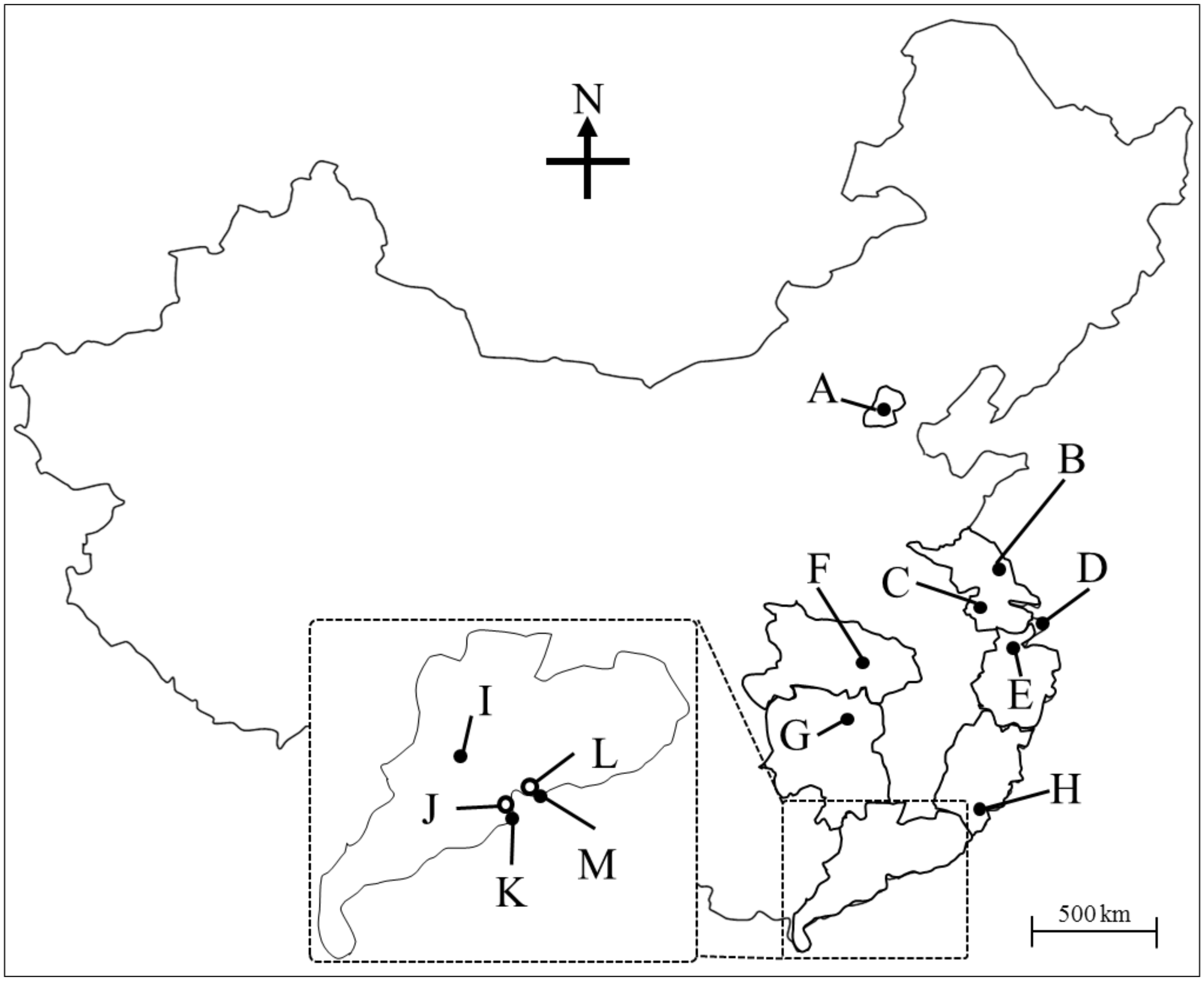

Supplemental Material, Figure S1. Locations of the 13 sampled cities in China.

See Supplemental Material, Table S2 for key to the location names and additional information about each location.

Supplemental Material, Table S3. Information about risk assessment for age-specific exposure scenarios and derivation of ADI/RSD and DWEL values.

Age-specific exposure scenario (U.S. EPA 2009)		Toxicological information for derivation of ADI or RSD				
**Age intervals**	**Daily water ingestion per body weight (mL/kg·day)[a]**	**Compound and corresponding ADI or RSD (µg/kg·day)**		**Toxicity endpoint**	**References**	**DWEL range throughout 12 age-intervals[b] (ng/L)**
1 to <3 months	205	Clarithromycin	0.2	MIC_{50} on *Peptostreptococcus spp.*	Citron and Appleman 2001	976 - 6452
3 to <6 months	159	Roxithromycin	0.4	MIC_{50} on *Eubacterum spp.*	Dubreuil 1987	1951 - 12903
6 to <12 months	126	Azithromycin	1.7	MIC_{50} on *Clostridium spp.*	Kitris et al. 1990	8293 - 54839
1 to <2 years	71	Tylosin	0.85	MIC_{50} on *Bifidobacterium spp.* and *Clostridium spp.*	FAO/WHO 2008	4146 - 27419
2 to <3 years	60	Sulfathiazole	50	Changes in thyroid tissue. Reference to the sulfamethazine which had a NOEL of 5 mg/kg for the thyroid effects in animal studies	Adopted from Schwab et al. 2005	243902 - 1612903
3 to <6 years	61	Sulfamethazine	1.6	Thyroid gland follicular adenoma in rats with tumor incidence data	Littlefield 1988	7805 - 51613
6 to <11 years	43	Sulfamethoxazole	130	Thyroid tumors in rats	Adopted from Schwab et al. 2005	634146 - 4193548
11 to <16 years	34	Thiamphenicol	0.9	Haemotoxic effects in rats and mice	Ando et al. 1997	4390 - 29032
16 to <18 years	31	Dimetridazole	0.006	Incidence of benign tumors of the mammary glands in rats, no slope factor	Lowe et al. 1976	27.8 - 184
18 to <21 years	35	Metronidazole	0.6	MIC_{50} for *Peptostreptococcus spp.*	Jokipii and Jokipii 1987	2927 - 19355

Age-specific exposure scenario (U.S. EPA 2009)		Toxicological information for derivation of ADI or RSD				
**Age intervals**	**Daily water ingestion per body weight (mL/kg·day)[a]**	**Compound and corresponding ADI or RSD (μg/kg·day)**		**Toxicity endpoint**	**References**	**DWEL range throughout 12 age-intervals[b] (ng/L)**
>21 years	39	Trimethoprim	4.2	MIC of the most sensitive species in human gut flora	Adopted from Schwab et al. 2005	20488 - 135484
>65 years	37	Metoprolol	14	Lowest therapeutic dose	Adopted from Schriks et al. 2010	68293 - 451613
		Clofibric acid	10	Reduction effect on serum cholesterol and triglycerides in patients with type III hyperlipoproteinemia	Adopted from Schriks et al. 2010	48780 - 322581
		Caffeine	150	Developmental effects (cleft palate) in rats exposed gestationally	Skalko et al. 1984	73171 - 483871
		Carbamazepine	0.3	Carcinogenicity in rats, no tumour data	Adopted from Bruce et al. 2010	1659 - 10968
		Diclofenac	67	No observable effects in mice exposed gestationally	Adopted from Bruce et al. 2010	326829 - 2161290
		Salicylic acid	26	Reproductive effects (increased duration of labor, maternal peripartum death)	Davis et al. 1996	126829 - 838710

[a] 95th-percentile values recommended;

[b] DWEL was calculated using the following equation: DWEL (ng/L)= [(ADI or RSD)×RSC_{DW}×BW×1000]/$IngR_{DW}$, where RSC_{DW}: relative source contribution of acceptable dose from drinking water, assumed to be 100% (most compounds) or 10% (caffeine only) for screening purposes; BW: body weight at each age-intervals; and $IngR_{DW}$: daily ingestion rate of drinking water per day. The highest level of each pharmaceutical in tap water was compared to the corresponding DWEL for each age interval to determine RQs at different life-stages.

Supplemental Material, Table S4. Occurrence (ng/L) and spatiotemporal distribution of 17 detected pharmaceuticals.

Sampling city (DI)[a]	and season	n		Clarithromycin	Roxithromycin	Azithromycin	Tylosin	Sulfathiazole	Sulfamethazine	Sulfamethoxazole	Thiamphenicol	Dimetridazole	Metronidazole	Trimethoprim	Metoprolol	Clofibric acid	Caffeine	Carbamazepine	Diclofenac	Salicylic acid
All	All	113	num[b]	8	8	8	4	1	6	10	13	22	45	2	1	31	98	26	2	37
			med[b]	6.7	2.8	7.0	6.4	<3.7[c]	9.4	8.0	17.8	6.9	1.8	10.2	<4.1	1.2	24.4	1.3	3.2	16.6
			max[b]	11.9	15.1	11.7	7.0	27.4	89.6	21.2	104.3	14.7	19.3	14.2	8.5	3.3	562.5	6.7	3.7	41.2
	Dry	67	num	6	8	7	4	1	5	8	8	12	27	2	1	26	55	18	2	19
			med	8.5	2.8	7.9	6.4	<3.7	11.2	6.6	33.6	7.8	2.2	10.2	<4.1	1.7	24.5	1.7	3.2	15.6
			max	11.9	15.1	11.7	7.0	27.4	89.6	21.2	104.3	14.7	19.3	14.2	8.5	3.3	562.5	6.7	3.7	41.2
	Wet	46	num	2	0	1	0	0	1	2	5	10	18	0	0	5	43	8	0	18
			med	1.8	<0.3	<0.3	<0.9	<3.7	<3.3	9.0	16.8	6.8	1.8	<5.2	<4.1	0.7	20.4	1.1	<1.2	19.0
			max	1.9	<0.3	1.2	<0.9	<3.7	5.5	9.1	26.5	9.7	8.4	<5.2	<4.1	1.2	79.9	1.8	<1.2	35.4
Beijing (0.4)	Dry	5	num	0	0	0	0	0	0	0	0	0	0	0	0	0	0	1	0	1
			med	<0.7	<0.3	<0.3	<0.9	<3.7	<3.3	<2.7	<5.2	<1.5	<0.4	<5.2	<4.1	<0.4	<3.8	<0.7	<1.2	<13.0
			max	<0.7	<0.3	<0.3	<0.9	<3.7	<3.3	<2.7	<5.2	<1.5	<0.4	<5.2	<4.1	<0.4	<3.8	1.9	<1.2	38.2
Yancheng (2.6)	Dry	5	num	0	0	0	0	0	0	0	0	0	3	0	0	0	5	5	0	0
			med	<0.7	<0.3	<0.3	<0.9	<3.7	<3.3	<2.7	<5.2	<1.5	1.3	<5.2	<4.1	<0.4	15.9	1.8	<1.2	<13.0
			max	<0.7	<0.3	<0.3	<0.9	<3.7	<3.3	<2.7	<5.2	<1.5	1.4	<5.2	<4.1	<0.4	18.3	2.4	<1.2	<13.0
Shanghai (2.9)	Dry	5	num	0	0	0	0	0	2	1	0	2	0	0	0	3	4	4	0	3
			med	<0.7	<0.3	<0.3	<0.9	<3.7	50.4	20.8	<5.2	5.0	<0.4	<5.2	<4.1	0.8	24.1	4.0	<1.2	15.6
			max	<0.7	<0.3	<0.3	<0.9	<3.7	89.6	20.8	<5.2	6.0	<0.4	<5.2	<4.1	1.0	62.7	6.7	<1.2	16.4

Sampling city (DI)[a] and season		n		Clarithromycin	Roxithromycin	Azithromycin	Tylosin	Sulfathiazole	Sulfamethazine	Sulfamethoxazole	Thiamphenicol	Dimetridazole	Metronidazole	Trimethoprim	Metoprolol	Clofibric acid	Caffeine	Carbamazepine	Diclofenac	Salicylic acid
	Wet	5	num	0	0	0	0	0	0	0	4	0	0	0	0	2	3	1	0	0
			med	<0.7	<0.3	<0.3	<0.9	<3.7	<3.3	<2.7	13.1	<1.5	<0.4	<5.2	<4.1	0.6	4.1	0.9	<1.2	<13.0
			max	<0.7	<0.3	<0.3	<0.9	<3.7	<3.3	<2.7	26.5	<1.5	<0.4	<5.2	<4.1	0.7	5.7	0.9	<1.2	<13.0
Hangzhou (6.0)	Dry	5	num[b]	4	4	4	4	1	1	2	4	2	5	0	0	5	5	4	0	1
			med[b]	10.1	2.6	9.8	6.4	<3.7[c]	<3.3	7.2	100.7	<1.5	1.8	<5.2	<4.1	1.1	434.5	0.8	<1.2	<13.0
			max[b]	11.9	3.0	11.7	7.0	27.4	7.6	11.6	104.3	1.8	2.6	<5.2	<4.1	3.2	562.5	1.0	<1.2	13.3
	Wet	5	num	2	0	1	0	0	0	0	0	0	2	0	0	2	5	0	0	2
			med	1.8	<0.3	<0.3	<0.9	<3.7	<3.3	<2.7	<5.2	<1.5	1.0	<5.2	<4.1	1.0	58.6	<0.7	<1.2	27.4
			max	1.9	<0.3	1.2	<0.9	<3.7	<3.3	<2.7	<5.2	<1.5	1.2	<5.2	<4.1	1.2	79.9	<0.7	<1.2	35.4
Nanjing (4.4)	Dry	5	num	0	0	0	0	0	0	0	4	5	5	0	0	0	5	0	0	4
			med	<0.7	<0.3	<0.3	<0.9	<3.7	<3.3	<2.7	23.4	11.6	15.5	<5.2	<4.1	<0.4	33.6	<0.7	<1.2	19.2
			max	<0.7	<0.3	<0.3	<0.9	<3.7	<3.3	<2.7	38.2	14.7	17.5	<5.2	<4.1	<0.4	53.1	<0.7	<1.2	41.2
	Wet	5	num	0	0	0	0	0	0	0	1	5	5	0	0	0	5	3	0	2
			med	<0.7	<0.3	<0.3	<0.9	<3.7	<3.3	<2.7	<5.2	8.9	6.0	<5.2	<4.1	<0.4	9.3	1.2	<1.2	14.2
			max	<0.7	<0.3	<0.3	<0.9	<3.7	<3.3	<2.7	16.8	9.5	8.4	<5.2	<4.1	<0.4	15.9	1.4	<1.2	15.1
Xiamen (1.0)	Dry	5	num	0	0	0	0	0	0	0	0	0	0	0	0	0	5	0	0	0
			med	<0.7	<0.3	<0.3	<0.9	<3.7	<3.3	<2.7	<5.2	<1.5	<0.4	<5.2	<4.1	<0.4	53.2	<0.7	<1.2	<13.0
			max	<0.7	<0.3	<0.3	<0.9	<3.7	<3.3	<2.7	<5.2	<1.5	<0.4	<5.2	<4.1	<0.4	67.9	<0.7	<1.2	<13.0
Wuhan	Dry	6	num	0	0	0	0	0	0	0	0	1	6	0	0	1	6	1	0	3
(3.0)			med	<0.7	<0.3	<0.3	<0.9	<3.7	<3.3	<2.7	<5.2	<1.5	1.6	<5.2	<4.1	<0.4	14.1	<0.7	<1.2	15.1

Sampling city (DI)[a] and season		n		Clarithromycin	Roxithromycin	Azithromycin	Tylosin	Sulfathiazole	Sulfamethazine	Sulfamethoxazole	Thiamphenicol	Dimetridazole	Metronidazole	Trimethoprim	Metoprolol	Clofibric acid	Caffeine	Carbamazepine	Diclofenac	Salicylic acid
			max	<0.7	<0.3	<0.3	<0.9	<3.7	<3.3	<2.7	<5.2	4.3	19.3	<5.2	<4.1	0.4	41.6	0.7	<1.2	16.8
Changsha (2.6)	Dry	5	num[b]	1	2	1	0	0	0	1	0	0	4	0	0	3	4	0	0	1
			med[b]	<0.7[c]	1.9	<0.3	<0.9	<3.7	<3.3	<2.7	<5.2	<1.5	3.0	<5.2	<4.1	<0.4	74.1	<0.7	<1.2	<13.0
			max[b]	1.0	2.9	1.1	<0.9	<3.7	<3.3	6.2	<5.2	<1.5	3.6	<5.2	<4.1	0.5	99.1	<0.7	<1.2	24.0
	Wet	5	num	0	0	0	0	0	0	0	0	0	4	0	0	0	4	0	0	1
			med	<0.7	<0.3	<0.3	<0.9	<3.7	<3.3	<2.7	<5.2	<1.5	1.5	<5.2	<4.1	<0.4	32.4	<0.7	<1.2	<13.0
			max	<0.7	<0.3	<0.3	<0.9	<3.7	<3.3	<2.7	<5.2	<1.5	1.8	<5.2	<4.1	<0.4	34.2	<0.7	<1.2	14.2
Guangzhou (5.2)	Dry	5	num	1	2	2	0	0	2	4	0	1	2	2	1	4	5	2	2	3
			med	<0.7	10.6	4.5	<0.9	<3.7	41.9	6.3	<5.2	<1.5	6.5	10.2	<4.1	2.3	23.4	2.6	3.2	16.0
			max	7.3	15.1	7.9	<0.9	<3.7	77.2	21.2	<5.2	11.6	10.4	14.2	8.5	3.0	185.7	3.3	3.7	19.6
	Wet	5	num	0	0	0	0	0	1	2	0	4	4	0	0	1	5	1	0	1
			med	<0.7	<0.3	<0.3	<0.9	<3.7	<3.3	9.0	<5.2	5.6	2.2	<5.2	<4.1	<0.4	20.1	<0.7	<1.2	<13.0
			max	<0.7	<0.3	<0.3	<0.9	<3.7	5.5	9.1	<5.2	9.7	5.1	<5.2	<4.1	0.7	41.8	1.8	<1.2	20.4
Zhuhai (1.9)	Dry	5	num	0	0	0	0	0	0	0	0	0	0	0	0	5	5	0	0	1
			med	<0.7	<0.3	<0.3	<0.9	<3.7	<3.3	<2.7	<5.2	<1.5	<0.4	<5.2	<4.1	2.1	24.3	<0.7	<1.2	<13.0
			max	<0.7	<0.3	<0.3	<0.9	<3.7	<3.3	<2.7	<5.2	<1.5	<0.4	<5.2	<4.1	2.5	28.5	<0.7	<1.2	14.2
	Wet	5	num	0	0	0	0	0	0	0	0	0	0	0	0	0	5	0	0	3
			med	<0.7	<0.3	<0.3	<0.9	<3.7	<3.3	<2.7	<5.2	<1.5	<0.4	<5.2	<4.1	<0.4	32.2	<0.7	<1.2	24.4
			max	<0.7	<0.3	<0.3	<0.9	<3.7	<3.3	<2.7	<5.2	<1.5	<0.4	<5.2	<4.1	<0.4	48.0	<0.7	<1.2	26.8
Macau (2.2)	Dry	5	num[b]	0	0	0	0	0	0	0	0	0	0	0	0	5	5	0	0	2

Sampling city (DI)[a] and season		n		Clarithromycin	Roxithromycin	Azithromycin	Tylosin	Sulfathiazole	Sulfamethazine	Sulfamethoxazole	Thiamphenicol	Dimetridazole	Metronidazole	Trimethoprim	Metoprolol	Clofibric acid	Caffeine	Carbamazepine	Diclofenac	Salicylic acid
			med[b]	<0.7[c]	<0.3	<0.3	<0.9	<3.7	<3.3	<2.7	<5.2	<1.5	<0.4	<5.2	<4.1	2.7	22.2	<0.7	<1.2	14.3
			max[b]	<0.7	<0.3	<0.3	<0.9	<3.7	<3.3	<2.7	<5.2	<1.5	<0.4	<5.2	<4.1	3.3	25.8	<0.7	<1.2	15.2
	Wet	5	num	0	0	0	0	0	0	0	0	0	0	0	0	0	5	0	0	5
			med	<0.7	<0.3	<0.3	<0.9	<3.7	<3.3	<2.7	<5.2	<1.5	<0.4	<5.2	<4.1	<0.4	37.7	<0.7	<1.2	20.3
			max	<0.7	<0.3	<0.3	<0.9	<3.7	<3.3	<2.7	<5.2	<1.5	<0.4	<5.2	<4.1	<0.4	46.7	<0.7	<1.2	26.0
Shenzhen (1.6)	Dry	5	num	0	0	0	0	0	0	0	0	1	2	0	0	0	4	1	0	0
			med	<0.7	<0.3	<0.3	<0.9	<3.7	<3.3	<2.7	<5.2	<1.5	1.1	<5.2	<4.1	<0.4	19.2	<0.7	<1.2	<13.0
			max	<0.7	<0.3	<0.3	<0.9	<3.7	<3.3	<2.7	<5.2	2.0	1.2	<5.2	<4.1	<0.4	71.9	0.8	<1.2	<13.0
	Wet	5	num	0	0	0	0	0	0	0	0	0	2	0	0	0	5	0	0	1
			med	<0.7	<0.3	<0.3	<0.9	<3.7	<3.3	<2.7	<5.2	<1.5	1.0	<5.2	<4.1	<0.4	51.4	<0.7	<1.2	<13.0
			max	<0.7	<0.3	<0.3	<0.9	<3.7	<3.3	<2.7	<5.2	<1.5	1.1	<5.2	<4.1	<0.4	64.1	<0.7	<1.2	13.2
Hong Kong (1.3)	Dry	6	num	0	0	0	0	0	0	0	0	0	0	0	0	0	2	0	0	0
			med	<0.7	<0.3	<0.3	<0.9	<3.7	<3.3	<2.7	<5.2	<1.5	<0.4	<5.2	<4.1	<0.4	8.4	<0.7	<1.2	<13.0
			max	<0.7	<0.3	<0.3	<0.9	<3.7	<3.3	<2.7	<5.2	<1.5	<0.4	<5.2	<4.1	<0.4	10.0	<0.7	<1.2	<13.0
	Wet	6	num	0	0	0	0	0	0	0	0	1	1	0	0	0	6	3	0	3
			med	<0.7	<0.3	<0.3	<0.9	<3.7	<3.3	<2.7	<5.2	<1.5	<0.4	<5.2	<4.1	<0.4	15.5	1.0	<1.2	16.7
			max	<0.7	<0.3	<0.3	<0.9	<3.7	<3.3	<2.7	<5.2	3.1	1.1	<5.2	<4.1	<0.4	20.2	1.2	<1.2	17.8

[a] DI: positive detection index, calculated by dividing total number of positive detections (levels ≥LOQ) of all compounds by the total sample number (both season) in each city [b] num: number of positive detection; med: median; max: maximum

[c] <LOQ

References

Alvanon. 2008. Chinese body measurement study – analysis reveals significant insight on Chinese size and shape for fashion industries. Available: http://www.alvanon.com/news/CHINASCAN.pdf [accessed 15 September 2011].

Ando J, Ishihara R, Imai S, Takano S, Kitamura T, Takahashi M, et al. 1997. Thirteen-week subchronic toxicity study of thiamphenicol in F344 rats. Toxicol Lett 91:137-146.

Bruce GM, Pleus RC, Snyder SA. 2010. Toxicological relevance of pharmaceuticals in drinking water. Environ Sci Technol 44:5619-5626.

Citron DM, Appleman MD. 2001. Comparative *in vitro* activities of ABT-773 against 362 clinical isolates of anaerobic bacteria. Antimicrob Agents Chemother 45:345-348.

Davis DP, Daston GP, Odio MR, York RG, Kraus AL. 1996. Maternal reproductive effects of oral salicylic acid in Sprague-Dawley rats. Toxicol Lett 84:135-141.

Dubreuil L. 1987. In-vitro comparison of roxithromycin and erythromycin against 900 anaerobic bacterial strains. J Antimicrob Chemother 20 (Suppl B):13-19.

FAO/WHO (Joint FAO/WHO Expert Committee on Food Additives). 2008. Evaluation of certain veterinary drug residues in food: 70th meeting report of the Joint FAO/WHO Expert Committee on Food Additives, Geneva:FAO/WHO.

Gaylor DW, Gold LS. 1998. Regulatory cancer risk assessment based on a quick estimate of a benchmark dose derived from the maximum tolerated dose. Regul Toxicol Pharmacol 28:222-225.

Gros M, Petrovic M, and Barcelo D. 2009. Tracing pharmaceutical residues of different therapeutic classes in environmental waters by using liquid chromatography/quadrupole - linear ion trap mass spectrometry and automated library searching. Anal Chem 81:898-912.

Jokipii AMM, Jokipii L. 1987. Comparative activity of metronidazole and tinidazole against *Clostridium difficile* and *Peptostreptococcus anaerobius*. Antimicrob Agents Chemother 31:183-186.

Kitris MD, Gold-stein FW, Miegi M, Acar JF. 1990. In-vitro activity of azithromycin against various Gram-negative bacilli and anaerobic bacteria. Journal of Antimicrobial Chemotherapy 25 (Suppl. A):15-18.

Leung HW, Minh TB, Murphy MB, Lam JCW, So MK, Martin M, et al. 2012. Distribution, fate and risk assessment of antibiotics in sewage treatment plants in Hong Kong, South China. Environ Int 42:1-9.

Littlefield N. 1988. Chronic toxicity and carcinogenesis study on sulfamethazine in Fischer 344 rats, unpublished report No. 420 from the National Center for Toxicological Research, Food and Drug Administration, Jefferson, Arkansas. Submitted to WHO by the U.S. Coordinator of the Codex Alimentarius, U.S. Department of Agriculture, Washington, D.C.

Lowe CY, Ingham B, Dale, EA. 1976. Dimetridazole (Emtryl): Tumourigenicity Study in Rats II. Unpublished report RES/2508 from the Pharmaceutical Research Laboratories, May and Baker Ltd., Dagenham, Essex, England. Submitted to WHO by Rhône-Poulenc Santé, Direction Scientifique, Paris, France.

NBSC (National Bureau of Statistics of China). 2009. Statistical data of environment in China. Available: http://www.stats.gov.cn/tjsj/qtsj/hjtjzl/hjtjsj2009/ [accessed 08 September 2011].

Schriks M, Heringa MB, van der Kooi MME, de Voogt P, Van Wezel AP. 2010. Toxicological relevance of emerging contaminants for drinking water quality. Water Res 44:461-476.

Schwab BW, Hayes EP, Fiori JM, Mastrocco FJ, Roden NM, Cragin D, et al. 2005. Human Pharmaceuticals in US surface waters: a human health risk assessment. Regul Toxicol Pharmacol 42:296-312.

Skalko RG, Poche PD, Kwasigroch TE. 1984. The toxicology of chemical interactions during pregnancy in the mouse: Caffeine and phenytoin. Toxicology 20:7-16.

U.S. EPA (U.S. Environmental Protection Agency). 2002. A review of the reference dose and reference concentration processes. Washington, DC: EPA. Available: http://www.epa.gov/raf/publications/pdfs/rfd-final.pdf [accessed 22 January 2013].

U.S. EPA. 2005. Guidelines for carcinogen risk assessment. Washington, DC: EPA. Available: http://www.epa.gov/cancerguidelines/ [accessed 22 January 2013].

U.S. EPA. 2009. Exposure factors handbook 2009 update (External Review Draft). Washington, DC: EPA. Available: http://cfpub.epa.gov/ncea/cfm/recordisplay.cfm?deid=209866 [accessed 22 January 2013].
